# Ataxin-2: a powerful RNA-binding protein

**DOI:** 10.1007/s12672-024-01158-y

**Published:** 2024-07-22

**Authors:** Lulu Li, Meng Wang, Lai Huang, Xiaoli Zheng, Lina Wang, Hongming Miao

**Affiliations:** 1 School of Basic Medical Science, Southwest Medical University, Luzhou, 646000 China; 2 Department of Pathophysiology, College of High Altitude Military Medicine, Army Medical University, Chongqing, 400038 China; 3 Department of Clinical and Military Laboratory Medicine, College of Medical Laboratory Science, Army Medical University, Chongqing, 400038 China

**Keywords:** ATXN2, Metabolism of RNA, RNA binding protein, Neurodegenerative diseases, Cancers

## Abstract

Ataxin-2 (ATXN2) was originally discovered in the context of spinocerebellar ataxia type 2 (SCA2), but it has become a key player in various neurodegenerative diseases. This review delves into the multifaceted roles of ATXN2 in human diseases, revealing its diverse molecular and cellular pathways. The impact of ATXN2 on diseases extends beyond functional outcomes; it mainly interacts with various RNA-binding proteins (RBPs) to regulate different stages of post-transcriptional gene expression in diseases. With the progress of research, ATXN2 has also been found to play an important role in the development of various cancers, including breast cancer, gastric cancer, pancreatic cancer, colon cancer, and esophageal cancer. This comprehensive exploration underscores the crucial role of ATXN2 in the pathogenesis of diseases and warrants further investigation by the scientific community. By reviewing the latest discoveries on the regulatory functions of ATXN2 in diseases, this article helps us understand the complex molecular mechanisms of a series of human diseases related to this intriguing protein.

## Introduction

The Short tandem repeats (STRs), commonly referred to as microsatellites, are repetitive DNA sequences spanning 2 to 6 nucleotides in length. These sequences are inherently unstable, prone to variations in the number of repeats, and their aberrant expansion is implicated in various pathological conditions [1]. Pathogenic mechanisms associated with STR amplification encompass loss of gene function, the formation of RNA foci enriched with repetitive motifs, the aggregation of polyglutamine proteins, and the non-canonical translation of toxic peptides stemming from repetitive sequences [2]. The overlap between Amyotrophic Lateral Sclerosis (ALS) and Frontotemporal Dementia (FTD) represents a significant genetic convergence point of clinical and pathological relevance [2].A prevalent genetic etiology for both ALS and FTD is the pathogenic amplification of STRs within the C9orf72 intron [2]. This amplification is discerned in approximately 7% of ALS cases and 6% of FTD cases, underscoring its pivotal role in these neurodegenerative disorders. While the C9orf72 STR expansion is the most established genetic basis for ALS and FTD, intermediate STR expansions within the Spinocerebellar ataxia (SCA) genes ATXN1 (SCA1) and ATXN2 (SCA2) have emerged as noteworthy contributors to ALS susceptibility [3].

Specifically, abnormal amplification of the CAG repeat sequence in ATXN2 gives rise to SCA2, an autosomal dominant neurodegenerative disorder primarily affecting the cerebellum, brainstem, and spinal cord [4]. The ATXN2 gene encodes a 140 kDa cytoplasmic protein [5] characterized by a trinucleotide CAG repeat sequence, which has been implicated in ALS-FTD [6] pathology. In the context of trinucleotide repeat expansion, ATXN2 adopts new toxic functions capable of driving disease progression [7]. These aberrantly amplified ATXN2 molecules accumulate and aggregate within the cell nucleus, where they may engage in intricate interactions with various proteins, exacerbating the pathological cascade [7].

The core of the pathogenesis of SCA2, ALS, and FTD, is the polyglutamine (polyQ) framework encoded by the CAG repeat sequence of ATXN2 [8]. Mutant ATXN2 proteins with polyQ expansions exhibit pronounced toxicity and contribute significantly to the disease process. Notably, the number of CAG repeats in the ATXN2 gene varies among individuals, with disease-associated alleles typically containing more than 34 repeats [9, 10]. Additionally, ATXN2 assumes a phenotypic modifier or genetic risk factor in ALS when the CAG repeat sequence falls within the range of 27 to 33 [11]. Furthermore, ATXN2 plays a pathogenic role as a phenotypic modifier in frontotemporal lobar degeneration (FTLD), with associations observed between CAG repeat amplification in ATXN2 and the age of onset of FTLD, as well as the manifestation of Parkinson's syndrome and psychotic symptoms [12].

Significantly, the genetic, biochemical, and neuropathological interactions between ATXN2 and TDP-43 strongly suggest the involvement of ATXN2 mutations in the pathogenesis of ALS and related conditions [13]. The dynamic nature of ATXN2 repeat sequences provides insights into the mechanisms through which ATXN2 mutations may contribute to ALS [12]. Amplification of intermediate-length ATXN2 CAG repeats is closely associated with ALS, and such expansions likely enhancing the stability or impeding the degradation of ATXN2, resulting in an elevated effective concentration of the protein [6]. Moreover, intermediate-length ATXN2 repeat sequences can trigger mislocalization from the nucleus to the cytoplasm, facilitating the mislocalization of TDP-43 into the nucleus [14].

Intriguingly, the inhibition of normal endogenous ATXN2 has been found to alleviate TDP-43 accumulation, revealing its role as a potential therapeutic target [15]. Furthermore, numerous studies have elucidated the capacity of ATXN2 to positively or negatively regulate the translation of specific mRNAs at the molecular level [16].

The intricate interplay between ATXN2 and human disorders has sparked significant interest in unraveling the molecular mechanisms that control its activities. Its significance in therapeutic development, ribonucleoprotein (RNP) assembly, cellular physiology, metabolic regulation, and animal behavior underscores the need for a comprehensive exploration. In this review, we will delve into the structural aspects of ATXN2, delineate its emerging functions, and summarize its implications in the onset and progression of diseases. Additionally, we will delve into the intricate mechanistic details that underlie these roles, shedding light on the multifaceted contributions of ATXN2 to the complex landscape of human disorders.

## Molecular structure and paralog of ATXN2

ATXN2, initially identified in humans due to its polyglutamine (polyQ) expansion linked to SCA2, a debilitating neurodegenerative disease, It is an evolutionarily conserved gene related to genetic susceptibility and predisposition to various diseases [17]. An exploration of ATXN2's structural domains across eukaryotes has unveiled the presence of ATXN2 and its paralog, ATXN2-like (ATXN2L), in all vertebrates, excluding birds [18].

Downstream of the Poly(Q) tract at the N-terminus of ATXN2, there are two key domains—the LSM domain and the LSM-associated domain (LSmAD). These domains play pivotal roles in various RNA processing activities, encompassing RNA modification, pre-mRNA splicing, mRNA decay, and separation [19]. The interplay of these domains extends to interactions with DDX6 and LSM12, which together control the post-transcriptional functions of ATXN2 [20]. Furthermore, ATXN2 encompasses a conserved proline-rich region and a C-terminal PAM2 motif (poly(A)-binding protein-associated motif 2), facilitating its interaction with poly(A)-binding protein (PABP) and thereby influencing RNA metabolism [21]. PABP itself features an MLLE (Mademoiselle) domain at its C-terminus, holding sway over poly(A) tail length, translation, mRNA degradation, and miRNA-dependent gene silencing [22].

**Fig. 1:**
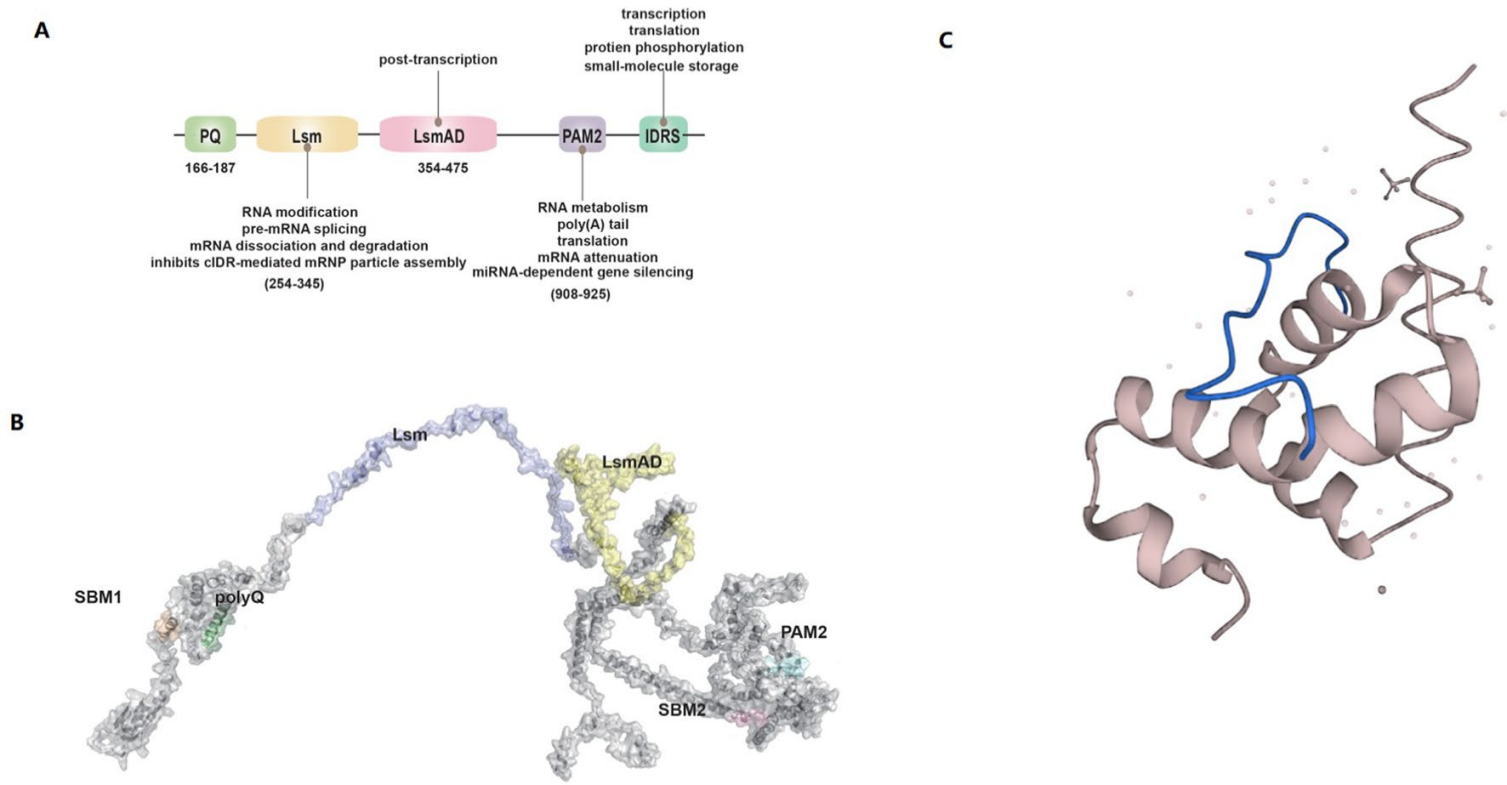
This is a figure. The Structure and Functions of ATXN2 Protein. **a** Human ATXN2 predominantly comprises structural domains including PQ, Lsm, LsmAD, PAM2, and IDRS. Lsm and PAM2 domains are associated with RNA metabolism, while LsmAD and IDRS domains play roles in transcription and translation processes. **b** Predicted architecture of the human ATXN2 protein model, generated using the Robetta online server.( http://robetta.bakerlab.org/fragmentsubmit.jsp). **c** Reposted to AlphaFold [30–32] (https://alphafold.com/entry/Q99700)

Notably, ATXN2 houses two intrinsically disordered regions (IDRs)—the intermediate IDR (mIDR), comprising polyglutamine and prion-like motifs, and the C-terminal IDR (cIDR) [23]. Despite their lack of a stable structure and high variability, IDRs exert significant control over various cellular processes, including transcription, translation, cellular signaling, protein phosphorylation, small-molecule storage, and the orchestration of large multiprotein self-assembly [24]. The cIDR domain contributes to the cohesion of mRNA ribonucleoprotein particles, while intriguingly, the Lsm domain inhibits cIDR-mediated ribonucleoprotein assemblyp [25](see Fig. 1).

Additionally, early investigations have identified essential elements for ATXN2 expression, including binding sites for exon splice enhancer proteins (ESTs) within the 5' untranslated region (UTR) of ATXN2 [26]. Manipulation of these elements, such as overexpression of EST1, has demonstrated the potential to modulate ATXN2 expression, hinting at the prospect of employing these regions as decoy targets for oligonucleotide-based therapeutic interventions.

In immortalized cell lines, ATXN2L, the paralog of ATXN2, is widely expressed in CD4-positive T-cell lymphomas and is associated with ATXN2L-JAK2 fusion [27]. This paralog, akin to ATXN2, is phylogenetically conserved [28] and plays a role in RNA surveillance. The functional domains of ATXN2 and ATXN2L bear strong resemblance, including the Lsm, LsmAD, and PAM2 domains, suggesting potential functional redundancy [18]. Nevertheless, it is worth noting that ATXN2L, lacking the poly(Q) expansion, exerts a more pronounced influence on cytoplasmic granule formation in mammalian cell culture [29].

## The role of ATXN2 in RNA metabolism

### Directly regulating translation and total mRNA stability

The involvement of ATXN2 in RNA metabolism is a multifaceted and complex process. Recent research has unveiled its role as an RNA-binding protein (RBP), capable of directly binding to over 4000 RNA molecules, with profound implications for both translation and overall mRNA stability [33].

Studies employing PAR-CLIP and high-throughput sequencing techniques have demonstrated that human ATXN2 can form direct interactions with target RNAs, obviating the need for the presence of poly(A)-binding protein (PABP) [34]. The LSM domain of ATXN2 enables it to recognize AU-rich regions within the 3' untranslated regions (UTRs) of target genes. By doing so, ATXN2 exerts a stabilizing effect on these target mRNAs, leading to an increase in their protein abundance [34]. Intriguingly, both the polyglutamine (Poly(Q)) tract within its N-terminus and the poly(A)-binding protein-associated motif 2 (PAM2) domain at the C-terminus contribute to ATXN2's capacity to enhance the stability and protein levels of target mRNAs [34]. Gene ontology (GO) analysis has further revealed that ATXN2 predominantly influences proteins involved in various RNA regulatory processes, such as 3' end processing, polyadenylation, and RNA splicing, among others [35].

**Fig. 2:**
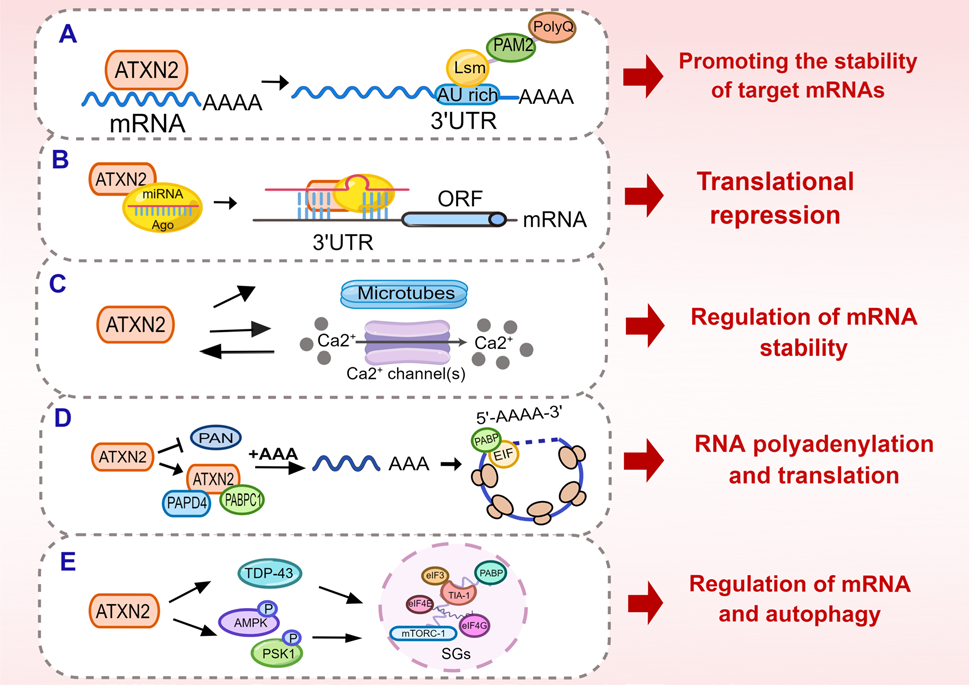
Signaling Pathways Downstream of ATXN2 Activation. **A** ATXN2 enhances the stability of target mRNAs and increases their protein abundance by recognizing AU-rich regions in the 3'UTRs of target genes through its LSM structural domain and other interactions. **B** ATXN2 interacts with AGO to mediate miRNA-induced silencing complex, thereby regulating translation repression. **C** ATXN2 influences mRNA stability by impacting microtubule stability and calcium channels. **D** ATXN2 may extend poly-A tails by inhibiting poly-A nuclease (PAN), interacting with PABPC1 or PAPD4, leading to RNA looping and promoting RNA polyadenylation. This, in turn, facilitates ribosomal recycling and translation activation. **E** ATXN2 localizes to stress granules (SG) and modulates RNA metabolism, autophagy, and associated pathways, such as mTOR signaling, through interactions with RNA-binding proteins (RBPs). This figure was generated using Figdraw (https://www.figdraw.com/)

Parallel studies employing PAR-CLIP have elucidated that ATXN2 shares some RNA target elements with HUR (Hu antigen R), an RNA-binding protein known to promote the stability and translation of target mRNAs through interactions with U-rich elements, including AU-rich elements (AREs) [36]. Notably, a subset of regulatory genes is jointly regulated by ATXN2 and HUR, with each protein binding to distinct U-rich motifs within specific 3' UTR locations [34] (see Fig. 2A).

### Complex role in translational regulation

The role of ATXN2 in translational regulation is characterized by its complexity and ability to modulate mRNA and protein levels in human and yeast cells. It's worth noting that in ATXN2 knockout (KO) mice [37], there is an overall reduction in protein synthesis. Paradoxically, some components related to ribosomal and translational control exhibit elevated mRNA and protein levels in ATXN2-KO mice. This phenomenon suggests that crucial components of the translational machinery may be upregulated as a compensatory mechanism in response to the global reduction in protein synthesis resulting from the absence of ATXN2 [38].

Furthermore, ATXN2 has been found to engage in a strong genetic interaction with AGO1, a key player in microRNA-mediated gene regulation [39] (see Fig. 2B). For instance, ATXN2 is required for the repression of translational reporters of genes like CaMKII (Ca^2+^-calmodulin-dependent kinase II) in projection neurons (PN) and local interneurons (LN) [39]. Additionally, ATXN2 plays a significant role in the regulation of mRNA and protein homeostasis during megakaryocyte formation [40].

Moreover, ATXN2 is involved in circadian rhythmic behavior and long-term memory in the nervous system of Drosophila through translational regulation. Biochemical analysis has shown that the ATXN2 protein is associated with translating polyribosomes, binds to RNA transcripts in both PAM2-dependent and PAM2-independent manners [41], and forms complexes with other known RBPs to play a role in diseases. Translation regulator 24 (TYF) utilizes ATXN2 to activate the translation of the rate-limiting circadian clock gene period (per), thus defining the post-transcriptional co-activator function of ATXN2 in the Drosophila circadian clock [42]. ATXN2-associated factors LSM12 and ME31B/DDX6 convert the ATXN2 complex into different post-transcriptional regulatory modes to control circadian rhythm and rhythmicity [43]: Firstly, LSM12 acts as a TYF-specific molecular adaptor for the ATXN2 complex, supporting TYF-dependent translational activity. Animal models with lsm12 defects demonstrate that the genetic, biochemical, and molecular pathways of ATXN2-LSM12-TYF maintain a 24-h periodicity through tyf-dependent PER translation. On the other hand, genetic analysis indicates that me31B and Not1 are involved in the per-independent clock function of ATXN2 to maintain high-amplitude rhythms of circadian rhythmic behavior and PDF cycling [43]. The circadian rhythm system of mice is affected by the lack of ATXN2, and ATXN2 has a strong influence on the transcriptional level of ROR-alpha [20], a transcriptional regulator of circadian rhythm in zebrafish. Recent studies have found that ATXN2 and ATXN2L undergo phase separation within cells, and this phase separation oscillates with the circadian rhythm. As RNA-binding proteins, ATXN2 and ATXN2L form droplets through phase separation that oscillate rhythmically and sequentially recruit and enrich a series of RNA-related biological processes. At the peak of oscillation, ribosomes and specific RNAs are recruited to promote the translation of key rhythmic proteins [44].

Post-translational modifications (PTMs) provide the basis for structural and functional diversity in the proteome and regulate fundamental cellular processes by modulating protein localisation, protein–protein interactions and biochemical properties [45]. It was found that ATXN2 is able to be phosphorylated by Cdk5, which controls ATXN2 protein abundance through proteasomal degradation in neurons, implying that Cdk5 activity is A therapeutic approach to SCA2 [46]. Parkin interacts with the terminals of ATXN2 to ubiquitinate normal and extended ATXN2, which together are involved in early-onset Parkinson's disease [47]. Parkin interacts with the terminals of ATXN2 to ubiquitinate normal and extended ATXN2, which together are involved in early-onset Parkinson's disease [45].

This intricate interplay between ATXN2 and various facets of RNA metabolism underscores its multifunctional role in the control of gene expression, with implications for both translational regulation and mRNA stability.

### ATXN2 and non-coding RNAs

ATXN2 has been shown to be able to protect genome integrity by binding to ncRNAs thereby hindering the deleterious accumulation of the nucleic acid structure of the R-loop, suggesting that we may be able to reduce R-loop formation by regulating the binding of ATXN2 to ncRNAs in certain diseases caused by the deleterious accumulation of the R-loop [38]. Previously, it was shown that ATXN2 is a protein capable of binding to the Me 31 B family of DEAD box deconjugating enzymes that are functionally related to Argonaute (Ago) and microRNAs (miRNAs) [40]. ATXN2 and miRNAs co-regulate synapse-specific long-term memory, and ATXN2 is required for miRNA-mediated translational repression.2 In yeast, the Ataxin-2 protein homologue Pbp1 binds non-coding RNA and inhibits the formation of RNA–DNA hybrids [48]. This binding is essential for maintaining genomic stability. Because of its important role in maintaining genome stability and regulating lifespan, Pbp1 may be a therapeutic target.

### *Affecting the stability of microtubules and Ca*^2+^*channels*

Microtubules and calcium ion (Ca^2+^) channels are pivotal players in the regulation of RNA and protein synthesis and stability [22]. In Drosophila, Urko del Castillo et al. demonstrated that the deletion of ATXN2 leads to increased microtubule stability. This effect, essential for organelle distribution and transport in Drosophila neurons, proves to be conserved across various cell types, both dividing and postmitotic, and is crucial for neural development, motility, and viability in Drosophila [44].

Furthermore, during the pre-onset phase of ATXN2-CAG100-Knock-In, a recent mouse model of ATXN2 pathology, there is a collective downregulation of several Ca^2+^-related proteins associated with transport or downstream intracellular signaling cascades collectively showed downregulation. The transcriptome profile of the disease indicates alterations in the expression of Ca^2+^ channels, transporters, and the CamKIV-modulated RNA splicing factor Khdrbs1/Sam68 due to ATXN2 pathology. Intriguingly, ATXN2's subcellular localization is influenced by cytoplasmic Ca^2+^ levels, and an imbalance in subcellular Ca^2+^ promotes ATXN2 re-localization into stress granules [49] (see Fig. 2C).

### Mediating cytoplasmic polyadenylation

Increasing evidence suggests that post-transcriptional polyadenylation of mRNAs positively regulates translation (see Fig. 2D). ATXN2 appears to stabilize its associated mRNAs by inhibiting mRNA decay induced by deadenylation [50]. Additionally, ATXN2 has been observed to lengthen the poly-A tail by attenuating poly-A nuclease (PAN) activity in yeast. Conversely, PABP enhances PAN activity, leading to a reduction in poly-A tail length [51]. Longer poly-A tails subsequently facilitate RNA looping through PABP's interaction with the 5'-cap-associating translation initiation factor (eIF4G), promoting ribosomal recycling and influencing the selection of selective polyadenylation sites [52]. Given that ATXN2 is significantly expressed in the hippocampus and hypothalamus [52], its mRNA may undergo selective polyadenylation, thereby promoting translation activity and neuroplasticity.

Transcriptional pulse-tracking analyses have suggested that ATXN2 promotes the post-transcriptional polyadenylation of target mRNAs, such as Cyclin D1 and TDP-43. This promotion is achieved by ATXN2 binding to PABPC1 [53]and the non-classical poly(A) polymerase PAPD4 through its IDRS region, recruiting PAPD4 to the target site. Notably, the polyadenylation signal, where mRNA cleavage and polyadenylation occur, is proximal to ATXN2's PAM2-dependent RNA-binding site in humans. It has been demonstrated that ATXN2 regulates the expression of target proteins by binding to the 3'-UTR of cyclin D1 and TDP-43 mRNAs. Subsequently, it was revealed that ATXN2 and PAPD4 collaboratively facilitate the polyadenylation of their target mRNAs to positively regulate gene expression [49]. However, it's worth noting that translation termination complexes, including eRF1-eRF3, Pan2-Pan3, and Caf1-Ccr4, can competitively interact with the polyadenylate-binding protein PABPC1 to mediate mRNA deadenylation [54]. The interactions between eRF3, Pan3, PAM2, and the C-terminal PABC structural domain of PABPC1 play crucial roles in these processes [55].

### As a component protein of stress granules

Stress granules (SGs) are dynamic, membrane-free compartments formed when RNA-binding proteins (RBPs) and RNA molecules temporarily aggregate in response to cellular stress, halting mRNA translation and redirecting the production of cytoprotective proteins [56]. ATXN2, typically localized in the cytoplasm, serves as a component protein of SGs and P bodies, significantly influencing the assembly and regulation of SGs [57]. During cellular stress, PABP and RNA from the rough endoplasmic reticulum (rER) undergo delocalization, along with ATXN2, to subcellular foci that serve as RNA–protein quality control centers, commonly referred to as SGs. These SGs can be distinguished by the presence of the marker protein TIA-1 (cytotoxic granule-associated RNA-binding protein) [58]. TIA-1, akin to ATXN2, translocates from the nucleus to promote the synthesis of cytosolic SGs during stress, binding to subsets of mRNAs and inhibiting their translation [59].

SGs primarily comprise the stalled 48S pre-initiation complex, which includes mRNA, small ribosomal subunits, eIF3, eIF4E, eIF4G, and PABP as core components [60]. All these components contain TIA-1, and ATXN2 knockdown leads to a decrease in the redistribution of TIA-1 from the nucleus to SGs, thereby impairing SG formation [60]. Moreover, ATXN2 has been shown to regulate translational repression in response to cellular stress induced by nutrient deprivation, and its expression increases in an mTOR signaling-dependent manner [61]. Key aspects of the mTOR pathway, including nuclear RNA splicing, RNA surveillance, ribosome biogenesis, cytoplasmic mRNA translation, and RNA degradation, are regulated by the ATXN2 gene [61]. Through the PI3K/mTOR signaling pathway, ATXN2 regulates eIF4E-binding protein 1 (4E-BP1) and interacts with numerous pre-initiation complex components during stressful conditions [61].

Recent discoveries have identified the double-stranded RNA-binding protein Staufen1 (STAU1) as a novel RNA-dependent interactor of ATXN2. Mutations in ATXN2 lead to abnormal autophagy and increased STAU1 abundance, which may contribute to the feedback regulation of autophagy by ATXN2 [57]. These findings suggest that ATXN2 within SGs can regulate RNA metabolism and autophagy by interacting with associated molecules or proteins (see Fig. 2E).

## ATXN2 and diseases

### ATXN2 and ALS

Amyotrophic lateral sclerosis (ALS) is a devastating neurodegenerative disease characterized by the progressive paralysis resulting from damage to upper and lower motor neurons. In the context of ALS research, ATXN2 initially emerged as a novel disease-associated gene through clinical investigations. However, the relationship between ATXN2 and ALS is primarily observed in ALS patients with medium-sized repeat sequences of ATXN2, which exhibit an abnormal distribution of ATXN2 in spinal cord neurons [62].

In the majority of ALS patients, ATXN2 exhibits more than 27 poly(Q) repeats [63]. Of note, the medium-length (Q) repeats of ATXN2 that are associated with ALS consist of CAA break repeats containing 1–3 CAA codon breaks, where CAA encodes glutamine. This configuration results in a pure poly(Q) structural domain within the protein repeat region [64]. Importantly, the stability of the CAG repeat sequence in ATXN2 is influenced by CAA disruption, leading to allelic instability and the pathogenic expansion observed in spinocerebellar ataxia type 2 (SCA2) [64].

Studies have demonstrated that the influence of ATXN2 repetitive sequences on ALS risk begins at 29 poly(Q) repeats, reaching a peak at 32 and 33 repeats. Remarkably, within the 29–33 intermediate repeat range, the loss of some Purkinje cells is noted in the cerebellum and spinocerebellar vermis of ALS patients. In mouse models of ALS, inhibiting ATXN2 expression significantly extends the lifespan of the animals, underlining the substantial role of ATXN2 in stress granule production and its contribution to aberrant TDP-43 cleavage in ALS [65].

In addition to ATXN2, a large hexanucleotide (GGGGCC) repeat amplification (HRE) in the C9orf72 gene has been identified as the most common genetic cause of ALS in both familial and sporadic Caucasian ALS patients. The presence of an intervening C9orf72 allele predisposes to massive amplification in offspring and disrupts the normal transcriptional activity of the C9orf72 promoter. This establishes the central role of C9orf72 in ALS pathogenesis [66].

Interestingly, ATXN2 also serves as a major modifier of ALS, with significant interactions observed between ATXN2 and pathologically expanded HRE-induced C9orf72 deletions, leading to ALS-frontotemporal dementia (ALS-FTD) pathogenesis. Intermediate ATXN2 repeat sequences may render C9orf72 HRE carriers more susceptible to ALS [67]. However, the potential interactions between non-pathological C9orf72 repeat sequences and ATXN2 repeat sequences in clinical contexts remain incompletely explored.

Notably, intermediate ATXN2 repeat sequences pose a risk factor for ALS patients with C9orf72 HREs and may modulate disease phenotypes, including clinical presentation and age of onset. Nevertheless, evidence in this regard is conflicting [68]. Recent research suggests that in a Chinese population, a cutoff of 31 repeats in ATXN2 is significantly associated with ALS risk, but when stratified by C9orf72 repeat length, ATXN2 repeat length showed no association with age of onset, delayed diagnosis or survival time [69]. Given the potential pathogenetic differences between C9orf72 HREs with reduced and increased expression, the role of ATXN2 in C9orf72 HRE carriers warrants reevaluation in ALS patients lacking C9orf72 HREs [69]. So, what is the clinical significance of ATXN2 intermediate repeat amplification in routine ALS clinical practice? Studies have reported varying incidence rates of ATXN2 intermediate repeat sequences in ALS patients [70]. The ALS clinic at Montreal's Neurological Institute Hospital (The Neuro), a leading provider of genetic testing for newly diagnosed ALS patients, incorporated the ATXN2 test into their offerings in October 2020 [71]. As a result, all newly diagnosed ALS patients now undergo extended testing for ATXN2 as part of standard clinical practice. To date, 61 newly diagnosed ALS patients have undergone ATXN2 genetic testing [71]. Following the introduction of ATXN2 intermediate repeat expansion testing as part of standard genetic testing, five patients have been identified: Four cases were due to retrospective screening of stored DNA samples and one case was due to prospective clinical testing [71]. Consistent with previous research, none of these patients had a family history of ALS or associated diseases, highlighting the imperative need for genetic testing in all ALS patients.

Genetics has long played a pivotal role in our understanding of ALS, and it is increasingly contributing to the development of targeted therapies. Consequently, the practice of genetic testing for ALS must evolve in tandem with emerging clinical trials. Routine ATXN2 testing is now established in clinical settings to identify ALS patients who may be eligible for targeted gene therapy trials. Retrospective screening of previously genetically tested patients using stored DNA samples within public healthcare systems alleviates the burden on financial and human resources [71].

Disease-modifying therapies targeting the ATXN2 poly(Q) intermediate repeat sequence present a promising strategy for treating ALS. Clinical evidence and research have highlighted ATXN2 as a key regulator of ALS survival. Clinical trials for gene therapy in ALS patients, including those with superoxide dismutase-1 (SOD1) mutations, C9orf72 hexanucleotide repeat amplification, ATXN2 trinucleotide amplification, fused in sarcoma (FUS) mutations, and sporadic cases without a known genetic cause, are currently underway. These trials are based on the multifaceted roles of ATXN2 in cellular processes, such as RNA metabolism, intrareceptor phagocytosis, RNA stability, and more. Specifically, ATXN2's involvement in stress granule formation and the induction of aberrant cleavage of TDP-43 by caspase 3 have been linked to ALS [72].

In 2018, studies demonstrated that induction of stress in HEK293T cells led to the binding of numerous nuclear cytoplasmic transit factors to ATXN2-containing stress granules. Additionally, the delivery of antisense oligonucleotides (ASOs) targeting ATXN2 to neuronal differentiated induced pluripotent stem cells (iPSCs) derived from C9orf72-ALS patients reversed the cytoplasmic mislocalization of nuclear proteins [57]. Groundbreaking in vivo work utilizing a rapidly progressing TDP-43 ALS mouse model showed that a single administration of ATXN2-ASO at birth resulted in sustained and significant reductions in ATXN2 mRNA levels, prolonged survival, and improved motor function. This approach not only benefits ATXN2-ALS patients but also offers a therapeutic avenue for a broader spectrum of ALS patients, as TDP-43 localization to ATXN2-dependent stress granules represents a common pathological endpoint. A Phase I clinical trial of ASO BIIB105, currently ongoing, is enrolling ALS patients, both with and without CAG repeat amplification in ATXN2 [57].

In summary, the burgeoning understanding of the role of ATXN2 in ALS pathogenesis has led to its integration into routine clinical practice for ALS diagnosis and treatment. Genetic testing for ATXN2 intermediate repeat amplification is becoming a standard procedure, enabling the identification of eligible patients for targeted gene therapy trials. As research continues to uncover the intricacies of ATXN2's involvement in ALS, promising avenues for therapeutic intervention emerge, bringing hope to individuals affected by this devastating disease.

### ATXN2 and SCA2

Recent research has expanded our understanding of ATXN2's role in neurodegenerative diseases, particularly in the context of spinocerebellar ataxia type 2 (SCA2). ATXN2 CAG repeat expansion and the presence of TDP-43 positive neuronal cytoplasmic inclusion bodies are shared molecular characteristics of both SCA2 and amyotrophic lateral sclerosis (ALS) [72]. However, despite these common molecular underpinnings, SCA2 and ALS are clinically distinct diseases. Interestingly, in some cases, ALS patients with expanded CAG repeats in the ATXN2 gene exhibit symptoms overlapping with both ALS and SCA2, particularly when the CAG repeats are expanded to a considerable length [73].

The repeated amplification of ATXN2 disrupts the ability of Purkinje cells to form stress granules, leading to cytotoxicity and neurodegeneration observed in both SCA2 and ALS. It's worth noting that, unlike in ALS, the repeat sequence of ATXN2 in SCA2 consists of a pure CAG repeat. This difference has led researchers to suggest that the length of CAG repeat amplification plays a role in determining the timing of clinical symptom onset in SCA2 and ALS [73].

Specifically, the expansion of the Poly(Q) structural domain of ATXN2 is a primary factor contributing to SCA2 when caused by the expansion of the CAG repeat sequence encoding glutamine in the disease gene. Studies examining 18 single nucleotide polymorphisms (SNPs) across the ATXN2 locus and their haplotypes have established that ATXN2 with 31–32 Poly(Q) repeats increases the likelihood of developing ALS. Furthermore, SNPs within the ATXN2/SH2B3 chromosomal region may exert an influence on the risk for a significant portion of ALS patients [74].

In addition, it has been shown that in the absence of mutant ATXN2 protein expression, the expATXN2 transcript is neurotoxic in a cell model with transducin beta-like protein 3 (TBL3), which is involved in rRNA processing and binds to expATXN2 and expanded Huntington's protein (expHTT) RNA in vitro. In both SCA2 and HD human brain tissues, rRNA processing is disrupted, resulting in the interruption of rRNA processing [75].

In conclusion, the varying composition of Poly(Q) repeats has distinct implications for disease manifestation. An investigation in Drosophila transduced with human ATXN2 protein revealed that ATXN2 encoded by pure CAG repeats exhibited toxicity in the retina and nervous system. In contrast, ATXN2 encoded by CAA-disrupted repeats or CAA repeats alone was expressed at comparable levels but did not induce toxic effects [76]. Notably, CAG-encoded ATXN2 protein forms aggregates in the eyes. The translation factor eIF4H, known for modulating the toxic effects of GGGGCC repeat sequences, has been found to impact the toxicity of ATXN2 protein encoded by the CAG gene. These findings suggest that ATXN2 encoded by pure CAG repeats exhibits different toxic properties compared to ATXN2 with disrupted CAA/G poly(Q) repeat structural domains, and the purity of the poly(Q) structural domain sequence is linked to disease pathogenesis [76]. Data derived from SCA2 cells and mouse models expressing mutant ATXN2 proteins support the central role of ATXN2 protein neurotoxicity in SCA2 pathogenesis. This neurotoxicity involves multiple cellular pathways, including messenger RNA (mRNA) maturation, translation, and endocytosis [77]. In all CUG/CAG diseases, repeat sequences containing mutant transcripts form RNA foci, contributing to RNA neurotoxicity and pathogenesis. It has been suggested that mutant ATXN2 transcripts with expanded CAG repeat sequences (expATXN2) induce neuronal cell death and interact with abnormalities in RNA-binding proteins (RBPs) involved in RNA metabolism in cellular and mouse models of SCA2, as well as in human SCA2 brains [75].

Furthermore, mutant ATXN2 tends to accumulate in the cerebellar tissue of SCA2 patients, disrupting calcium homeostasis in affected neurons and displaying features of functionally acquired mutations [78]. Overexpression of ATXN2 mutants leads to SCA2-associated neuropathological and behavioral abnormalities, neuroinflammation, cell death, and affects the expression of LC3B and SQSTM1 proteins in the autophagy pathway of SCA2 [79]. Oligomeric ATXN2 and oxidative stress affect autophagic clearance in SCA2 cells [80].

When the N-terminal poly(Q) domain of proteins containing poly(Q) expands, they are closely related to the development of neurodegenerative diseases. Regarding ATXN2, early research detected poly(Q) containing ATXN2 fragments when using brain extracts from patients with SCA2. These fragments underwent specific N-terminal proteolytic cleavage, and this cleavage did not depend on the length of poly(Q). This specific proteolysis may alter the normal function of ATXN2 or produce toxic protein fragments, leading to neuronal damage and degenerative changes. However, the specific mechanism of action and how these proteolytic products affect the pathological process of SCA2 still require further research to clarify [81].

Previously in SCA2, it was recently demonstrated that mutant ATXN2 can be selectively down-regulated by FBXW8 (a subunit of the ubiquitin ligase complex), and that both FBXW8 and PARK2 (an E3 ubiquitin ligase) are recruited to mutant ATXN2 aggregates in both cellular and animal models [82]. This suggests that there may be damage to the ubiquitin proteasome system (UPS) that, if persistent, could lead to toxicity and neurodegeneration.Failure of the UPS may also lead to splitting of the mutant proteins into smaller, more toxic fragments, which has been implicated in the pathogenesis of a wide range of neurodegenerative traits involving the accumulation of aberrant proteins [83]. In SCA2, artificial inhibition of UPS produced 70 kDa fragments in cells transfected with mutant ATXN2, but not in cells transfected with the WT form. In addition, a 42 kDa fragment was detected in human postmortem brain samples in response to poly(Q)-specific 1C2 antibodies [84]. Interestingly, this fragment (probably N-terminal given the location of the poly(Q) region) was detected in both SCA2 patients and cellular models of the disease, but not in control samples.

In addition,some scholars concluded that the cytoplasmic aggregation pattern of mutant ATXN2 is prevalent in the early stages of SCA2, while the presence of nuclear inclusion bodies is associated with the final stage. According to these and other studies [85], cytoplasmic ATXN2 particles may be the first aggregating species in SCA2, actively contributing to pathogenesis by disrupting essential cellular mechanisms [86].

### ATXN2 and metabolism-related diseases

Research on ATXN2 has extended to its involvement in metabolism-related diseases, yielding valuable insights into its multifaceted roles. Some researchers have discovered that the knockout of the ATXN2 gene in mice leads to notable alterations in body weight, insulin sensitivity, and fertility [87]. In ATXN2 knockout (ATXN2-KO) mice, significant changes have been identified in the expression levels of various lipid metabolism-related factors, including sphingolipids, cholesterol homeostasis, sphingomyelinase A-SMase, PPARδ (a transcriptional lipid regulator), and BLBP (a fatty acid-binding protein). These findings suggest a potential role for ATXN2 in lipid metabolism [88].Recent investigations using proteomic and metabolomic approaches in ATXN2 KO mice further corroborated the impact of ATXN2 on lipid metabolism. Notably, the downregulation of short-chain-specific acyl-coenzyme A dehydrogenase (Acads), an enzyme involved in the oxidation of fatty acids, was observed in ATXN2-deficient mice. This downregulation could promote the formation of fat droplets in the liver. Moreover, key metabolic pathways, including branched-chain amino acid (BCAA) metabolism, fatty acid metabolism, and the citric acid cycle, were significantly downregulated in the liver of ATXN2 KO mice [89].

In contrast, mice engineered with the mouse ATXN2 gene carrying CAG100 amplification display transient weight loss, brain atrophy, motor deficits, and impaired synthesis of acetyl coenzyme A and aspartate by neuronal mitochondria. Additionally, these mice exhibit altered levels of metabolites, including NAA (which is essential for myelin production), and an increase in blood cholesterol levels [90]. Subsequent studies in ATXN2-CAG100-KIN mice revealed persistent reductions in various ceramides in the cerebellum and spinal cord, with a significant increase in sphingomyelin levels in the severely affected spinal cord. These findings suggest abnormalities in ceramide-sphingomyelin metabolism [91].

Further insights into the role of ATXN2 in metabolism come from observations in the cerebellum of ATXN2 KO mice. These mice exhibited the downregulation of key factors involved in calcium homeostasis, including transcription factor Rora, transporter proteins ITPR1 and Atp2a2, and regulator Inpp5a. This downregulation, some of which occurred early in the proximal cerebellum, has been linked to Purkinje cell calcium-mediated excitation and ATXN2-induced neurotoxicity, highlighting the physiological roles and protein interactions of ATXN2 [92].

The mTOR (mammalian target of rapamycin) pathway plays a pivotal regulatory role in various cellular activities, such as protein and lipid synthesis, cell size and growth, transcription, and translation [93]. This pathway consists of two major protein complexes: mTOR complex 1 (TORC1) and complex 2 (TORC2) [94]. The human homolog of ATXN2, ATXN2, controls the mTOR pathway by targeting molecular targets downstream of AMPK and upstream of ribosomal protein S6 kinase and TORC1 [95]. Downregulation of ATXN2 in animals results in accelerated growth and increased fat accumulation, leading to sterility. Conversely, upregulation of ATXN2 inhibits the mTOR pathway. Furthermore, in mouse embryonic fibroblasts lacking ATXN2, increased phosphorylation of translation regulators 4E-BP1 and ribosomal protein S6 in the PI3K/mTOR pathway has been observed. In human neuroblastoma cells, ATXN2 transcriptional suppression via mTOR increases significantly following food restriction [61].

Intriguingly, ATXN2 in the hypothalamus appears to be a critical determinant of body weight, insulin sensitivity, and the expression of biological clock genes. Notably, hypothalamus-specific overexpression of ATXN2 prevents high-fat diet (HFD)-induced obesity and insulin resistance [96]. Moreover, the reconstitution of ATXN2 in ATXN2 KO mice ameliorates metabolic dysfunction without affecting body weight. These findings highlight the potential role of ATXN2 in regulating the biological clock by influencing biological clock genes, shedding light on a novel mechanism in metabolic regulation [96].

In summary, ATXN2's multifaceted involvement in lipid metabolism, calcium homeostasis, and the mTOR pathway underscores its significance in metabolism-related diseases, offering potential avenues for therapeutic exploration and the development of new treatments.

### ATXN2 and Glaucoma

Glaucoma is a progressive optic neuropathy characterized by the gradual deterioration of retinal ganglion cells and the retinal nerve fiber layer (RNFL), leading to associated visual field abnormalities [97]. The primary risk factor for glaucoma is elevated intraocular pressure (IOP), which is also the only modifiable risk factor [98]. Recent studies have linked high IOP in primary open-angle glaucoma (POAG) to single nucleotide polymorphisms in the ATXN2 gene [99]. ATXN2 is predominantly localized in retinal ganglion cells and is expressed in the ciliary body and trabecular meshwork, with a consistent expression pattern observed in both mouse and human retinas [100].

### ATXN2 and Huntington's disease

Huntington's disease (HD) results from the expansion of the CAG triplet in the Huntington gene (HTT), leading to an expanded polyglutamine poly(Q) repeat (mHTT) that aggregates within cells, contributing to disease pathology [101]. Studies have indicated that the structural PAM2 domain of ATXN2 exhibits enhanced toxicity toward mHTT. ATXN2 acts as a strong, dose-dependent mediator of mHTT effects, potentially promoting mHTT aggregation and toxicity by activating the transcription factor CrebA. Furthermore, ATXN2 RNA interference (RNAi) may partially mitigate pre-degenerative neuronal dysfunction induced by mHTT, and CrebA knockdown has a similar effect in reducing mHTT toxicity. Conversely, CrebA overexpression counteracts the impact of ATXN2 RNAi, highlighting a unique molecular mechanism by which ATXN2 regulates mHTT toxicity [102].

In a Drosophila model of Huntington's disease, the cIDR of ATXN2 has been found to promote Huntington's protein aggregation and neurodegeneration, indicating its essential role in protein inclusion body formation. Since HTT is not a known component of ATXN2-containing RNP (ribonucleoprotein) particles, and HTT-poly(Q) inclusions do not contain additional RNP particle components, the precise function of ATXN2-dependent macromolecular assembly in HD development remains a challenge to decipher. While researchers have proposed three broad mechanisms to explain these observations, further studies are needed to fully elucidate the role of ATXN2 in Huntington's disease [103].

### ATXN2 and frontotemporal lobar degeneration (FTLD)

Frontotemporal lobar degeneration (FTLD) is a form of dementia characterized by behavioral disturbances and language problems [104]. FTLD, along with ALS, is classified as a TDP-43 proteinopathy, characterized by the aggregation of TDP-43 in brain aggregates [105]. TDP-43 is a heterogeneous nuclear ribonucleoprotein (hnRNPs) with essential roles in RNA regulation, and its accumulation, mislocalization, and aggregation contribute to neurodegenerative diseases [106].

Originally, ATXN2 was primarily investigated in the context of ALS, but subsequent research has highlighted its pathogenic involvement in FTLD, where mutant ATXN2 leads to the formation of poly(Q) bundles and aberrant modification of TDP-43 [107]. The moderate repeat amplification sequence of ATXN2 is correlated with the age at which initial FTLD symptoms appear. Silencing ATXN2 genetically enhances the translational machinery through the phosphorylation of RPS6, although it decreases the overall rate of protein synthesis. Recent studies have shown that endogenous ATXN2 suppression can reduce TDP-43 accumulation, suggesting a potential therapeutic target for TDP-43 proteinopathy [107].

By crossbreeding ATXN2 knockout mice with TDP-43 transgenic mice, researchers demonstrated that ATXN2 deficiency significantly reduces TDP-43 accumulation, prolongs survival, and improves motor performance. Additionally, the administration of antisense oligonucleotides targeting ATXN2 significantly extends the lifespan of TDP-43 transgenic mice. These findings suggest that ATXN2 in the brains of FTLD-TDP patients may be downregulated as a secondary response to mitigate the neurotoxic effects of TDP-43 aggregates [107].

### ATXN2 and Zika/COVID-19 Virus

Stress responses, which activate global translational pauses and induce the formation of stress granules (SGs), represent an initial line of defense against viruses in mammalian cells [108]. SGs are formed through the phosphorylation of eukaryotic initiation factor 2α (eIF2α) and play a role in maintaining RNA homeostasis under stressful conditions. SGs can attract specific viral proteins, including those from positive-stranded RNA viruses such as SARS-CoV-2 and Zika virus (ZIKV) [108]. While the connection between ATXN2 and SARS-CoV-2 replication based on their association with stress granules has not been definitively established, it represents a promising direction for future research.

Preliminary studies have shown that ZIKV, a single-stranded positive RNA virus, induces apoptosis upon infecting cells, indirectly leading to the phosphorylation of eIF2α and global translation inhibition. ZIKV RNA and coat protein hinder SG assembly by targeting key nucleation factors of SGs [108]. Recent research has demonstrated that ATXN2 elimination reduces ZIKV RNA and viral titers, effectively controlling ZIKV gene expression. Additionally, ATXN2 regulates ZIKV gene expression in hepatocellular cancer cells [109]. While the relationship between ATXN2, ZIKV, and SGs remains to be fully elucidated, these findings contribute to our understanding of the pathological mechanisms underlying ZIKV congenital disease, considering ATXN2's role in aspects of SG formation and altered RNA homeostasis, including RNA splicing, stability, and translation.

Furthermore, ATXN2 has emerged as a potential risk gene for the novel coronavirus (COVID-19), and its effects are shared with asthma at the genome-wide level [110].

### ATXN2 and immunological effects

While specific studies on ATXN2's function in immunity are limited, a notable connection has been established between a missense mutation in the SH2B adaptor protein 3 (SH2B3) and an ATXN2 varian [111]t. Another variant of ATXN2 is highly associated with a missense variant (R262W) within the SH2B3 gene. The non-ancestral allele W262 increases the levels of T lymphocytes and the subset of helper CD4 + T cells, with similar effect sizes, and is positively correlated with many autoimmune diseases as well as hypertension and related pathologies [112].The presence of TAC risk haplotype in the ATXN2-SH2B3 locus may increase the risk of thrombosis in antiphospholipid antibodies (aPLA) carriers [113].

In conclusion, although the precise roles of ATXN2 in immunity remain unclear, ATXN2 could potentially serve as a valuable marker for certain immune-related diseases and may provide insights into new therapeutic targets. Regrettably, ATXN2 has not received significant attention in subsequent studies on immune aspects. Consequently, ATXN2 remains a relatively novel and multifunctional molecule in immunoregulation, warranting further in-depth investigation into the relevant molecular mechanisms.

### ATXN2 and Machado-Joseph disease

Machado-Joseph disease, also known as spinocerebellar ataxia type 3 (MJD/SCA3), represents the most prevalent autosomal dominant ataxia worldwide [114]. MJD is attributed to the expansion of a polyglutamine poly(Q)-encoding CAG repeat within the ATXN3 gene [115]. Recent findings have indicated that mutant ATXN3 with expanded poly(Q) tracts contributes to transcriptional impairment, mitochondrial dysfunction, dysregulation of oxidative stress mechanisms, and apoptosis in MJD [116]. Some of these alterations, including transcriptional dysregulation, have been observed in blood samples from MJD carriers.

Studies have shown that in patients and animal models with Machado-Joseph disease, ATXN2 levels are reduced, while the accumulation of ATXN3MUT drives ATXN2 from the cytoplasm into the nucleus. The aggregation of ATXN3MUT in the nucleus is associated with a significant decrease in ATXN2 mRNA and protein levels, as well as ATXN2 aggregation, further reducing the level of this protein in the cytoplasm [116]. In a transgenic mouse model of Machado-Joseph disease, the decrease in ATXN2 levels may cause translational dysregulation by releasing PABP from overly active protein translation. Additionally, ATXN2 interacts with PABP through the PAM2 motif to reduce ATXN3 translation, alleviating motor and cerebellar deficits [116].

The age at onset of Machado-Joseph disease varies among different racial populations. In European populations, the age at onset in MJD is influenced by CAG repeat sequences in ATXN2, ATN1, and HTT. In a study of the Chinese population, in addition to CAG repeats in ATXN2 and ATXN3, a significant association was found with a functional SNP in ATXN2, rs7969300 [117]. It is speculated that rs7969300 may regulate the age at onset by affecting the stability of ATXN2, which subsequently interferes with the interaction between ATXN3 and ATXN2 [117].

### ATXN2 and cancers

The relationship between ATXN2 and tumorigenesis has been a focal point of recent research, mostly reporting positive correlations as summarized in Tables 1 and 2. In brief:

**Table 1:** ATXN2 expression in various cancers

Cancer type	ATXN2 expression	Molecular/cellular finding	Clinical findings	Reference no.
Stomach	↑	↑mRNA, ↑ATXN2 protein	↓survival	[122]
Esophagus	↑	↑mRNA, ↑ATXN2 protein	↑proliferation, ↑migration,	[118, 126]
			↑invasion,↓survival	
Ovaries	↑	↑methylation	–	[125]
Colon	↓	↓mRNA	–	[123]
Pancreas	↑	↑mRNA	↑proliferation, ↑migration	[119]
neuroblastoma	↑	↑ATXN2 protein	↑apoptosis	[120]

↑: increased,↓: decreased

The table shows the correlation between ATXN2 expression and molecular/cellular outcomes as well as clinical consequences in different cancers

**Table 2:** Possible mechanisms involving ATXN2 during tumorigenesis

Cancer/cell type	Associated molecules	Cancer-related function	Possible ATXN2 action	Reference no.
Esophageal squamous cell carcinoma	TNFR1	↑cell proliferation,	bind to m6A-methylated TNFR1 and mediate translation of TNFR1	[118]
		↑cell migration,		
		↑invasion		
Pancreatic adenocarcinoma	miR-873-3p/ LINC00941	↑cell proliferate, ↑metastasize	Combine with miR-873-3p	[119]
Neuroblastoma cells	MYCN/ IFNγ	↑cell apoptosis	Change in ATNX2 protein expression/ activate caspase 8	[120]
Colon tumors	ZBRK1	↓Transcription	Interacting with ZBRK1 and regulating SCA2 gene transcription	[123]
Gastric cancer	SP1/PI3K/AKT	↑cell apoptosis, ↑chemoresistance ↑immune escape	↑PD-L1 ↓CD8 + T Cell cytotoxicity, activate PI3K/AKT	[122]

The table shows the possible roles for ATXN2 in candidate signaling pathways and the probable actions

In esophageal squamous cell carcinoma (ESCC), ATXN2 has been found to be significantly elevated. ATXN2 functions as a critical protein involved in m6A methylation in ESCC, enhancing the translation of TNFRF1A by binding to m6A-modified TNFRF1A mRNA. Elevated TNFRF1A levels are associated with reduced survival in ESCC patients. The METTL3-m6A-TNFRF1-ATXN2 axis plays an oncogenic role in ESCC through the MAPK/NF-κB signaling pathway [118]. In pancreatic adenocarcinoma (PAAD), ATXN2 is upregulated in PAAD tissues and exhibits negative associations with miR-873-3p levels but positive associations with LINC00941 levels. LINC00941 stimulates PAAD cell proliferation and metastasis by competitively binding to miR-873-3p, leading to the upregulation of ATXN2 [119]. In neuroblastoma, ATXN2 overexpression sensitizes neuroblastoma cells to apoptosis. Neuroblastoma tumors with amplified MYCN contain significantly less ATXN2 protein compared to tumors without amplified MYCN [120]. ATXN2 plays a crucial role in regulating the susceptibility of neuroblastoma cells to apoptotic stimuli both in vitro and in vivo [120]. In colon tumors, ATXN2 acts as a co-regulator of ZBRK1, enhancing its own transcription (SCA2 gene). Elevated ZBRK1 levels result in increased ATXN2 levels, while transcriptional and protein interference of ZBRK1 leads to reduced ATXN2 levels. Significantly, ATXN2 is reduced in colon tumors with low ZBRK1 transcripts [121]. The PI3k/AKt pathway is a key regulator of tumour pathology, and activation of the PI3K/AKT signaling pathway promotes tumour cell proliferation, inhibits apoptosis, and is closely associated with tumour invasion and metastasis.In gastric cancer (GC) [122], ATXN2 expression influences the expression of cancer-associated gene products, thereby accelerating cancer progression. In gastric cancer, SP1 transcriptionally regulates ATXN2 expression, activating the PI3K/AKT signaling pathway, which contributes to ATXN2's anti-apoptotic and chemoresistant properties in gastric cancer. Upregulated ATXN2 expression also leads to increased PD-L1 expression, impacting gastric cancer immunotherapy [122]. Moreover, the missense mutation (rs3184504) in SH2B3 is associated with breast cancer [123]. The highly correlated variation in the SH2B3/ATXN2/ BRAP locus (including rs3184504) is related to the age of parental death [124], suggesting that ATXN2 may also play a regulatory role in breast cancer.

In other cancers, such as ovarian cancer, ATXN2 is linked to specific methylation profiles [125]. In esophageal adenocarcinoma, ATXN2 significantly correlates with patients' overall survival [126]. Additionally, inhibiting ATXN2 through siRNA increases the efficiency of nanoparticle delivery to tumor cells, making ATXN2 a potential reference for nanoparticle delivery prediction in cancer cells [127].

## Discussion

**Fig. 3:**
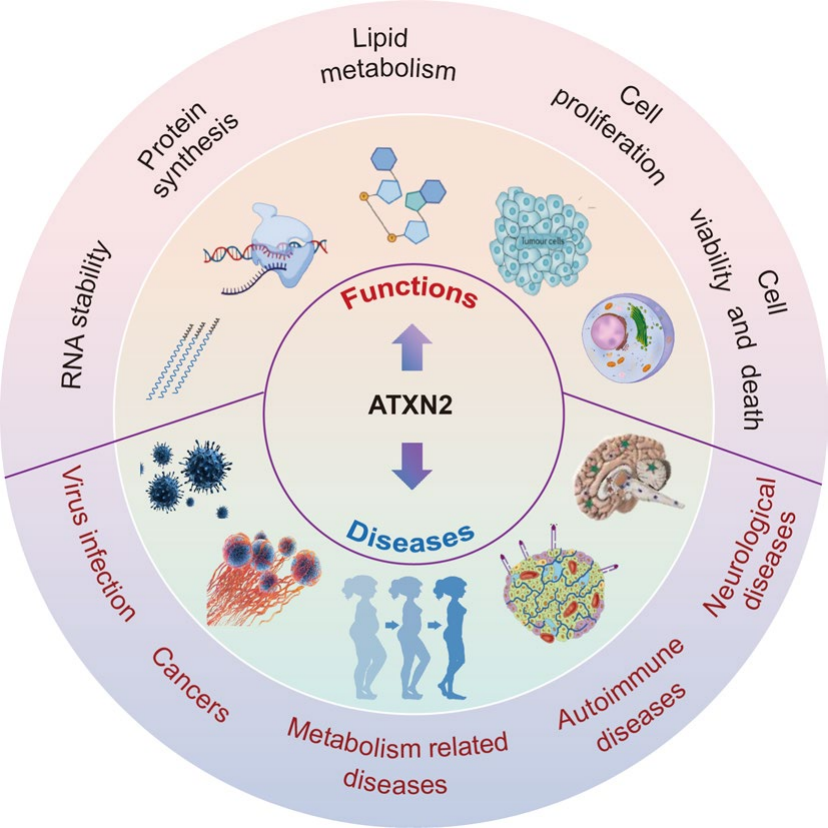
Cellular and Pathological Functions of ATXN2. This schematic provides an overview of the multifaceted functions of ATXN2 at both cellular and pathological scales. This figure was generated using Figdraw (https://www.figdraw.com/)

A plethora of evidence from numerous studies highlights the diverse range of functions associated with ATXN2 in various diseases (Fig. 3). Initially, researchers observed that the length of poly(Q) repeat amplification in ATXN2 is implicated in the pathogenesis of ALS, SCA2, and Parkinson's disease, and that the sequence encoding the poly(Q) repeat varies across these diseases [76]. SCA2 is characterized by a pure CAG repeat, whereas the CAG repeat in ALS and Parkinson's disease often contains interruptions with the poly CAA codon [26]. Notably, RNA structures with pure CAG repeats form hairpin structures [128], capable of sequestering RNA-binding proteins (RBPs), thereby affecting protein function and other cellular processes [129]. In contrast, RNA interrupted by CAA sequences exhibits different structural properties and may not compartmentalize proteins to the same extent [130]. ALS is primarily associated with cytoplasmic aggregates of TDP-43, a key pathological protein in neurodegeneration [131]. Intriguingly, a study found that moderate repeat amplification of the ATXN2 gene in mice led to a reduction in TDP-43 aggregates, resulting in improved survival and motor function [132]. This suggests that the length of the ATXN2 CAG repeat may hold therapeutic potential for ALS cases characterized by TDP-43 aggregates. However, it remains a major challenge to analyze the differential effects of CAG repeat length and the abundance of TDP-43 aggregates among patients with varying disease severity and age.

Continuing research efforts have unveiled ATXN2's interaction with various factors associated with tumorigenesis, providing novel avenues to explore the molecular mechanisms underlying cancer. For instance, ATXN2 has been shown to enhance the translation of TNFRSF1A by binding to m6A-modified TNFRSF1A mRNA, leading to the activation of NF-κB and MAPK pathways, thereby promoting esophageal squamous cell carcinoma (ESCC) tumorigenesis and progression [133].

ATXN2L, a homolog of ATXN2 lacking an abnormal poly(Q) expansion, is phylogenetically conserved and functions as an RNA monitor through its RNA-binding Lsm and LsmAD motifs, as well as its pabpc1-binding PAM2 motif. ATXN2L is also implicated in disease development. For example, in gastric cancer, ATXN2L expression is upregulated by EGF, which activates the PI3K/AKT signaling pathway, promoting epithelial-mesenchymal transition (EMT), migration, and invasion, all associated with a poor prognosis in gastric cancer. In contrast, in oxaliplatin-treated gastric cancers, ATXN2L is involved in Schwann cell assembly, contributing to drug resistance, recurrence, and disease progression in gastric cancer. Additionally, a highly conserved de novo heterozygous mutation in ATXN2L has been suggested to play a role in large head malformation developmental delay syndrome, although the exact mechanism necessitates further experimental validation [28]. In diabetic peripheral neuropathy (DPN), jatrorrhizine treatment has demonstrated the ability to reduce the thresholds for thermal and mechanical stimulation while increasing nerve conduction velocity. Furthermore, jatrorrhizine increases the expression of myelin-related proteins (MBP, MPZ, and PMP22). Histone deacetylase 3 (HDAC3) is a critical target for jatrorrhizine-induced enhancement of myelin formation. HDAC3 interacts with ATXN2L, thereby antagonizing the NRG1-ErbB2-PI3K-AKT signaling axis. Thus, jatrorrhizine modulates the NRG1-ErbB2-PI3K-AKT pathway by inhibiting the recruitment of ATXN2L by HDAC3, ultimately improving myelin sheath formation in DPN mice [134].

CAG repeat amplification of the ATXN2 gene is a causative factor in SCA2 [74].Therapeutic approaches may involve strategies to target this gene mutation, such as targeting ATXN2 mRNA using antisense oligonucleotides (ASOs) to reduce the production of aberrant ATXN2 protein. In mouse models of SCA2, this approach has been shown to significantly reduce ATXN2 mRNA and protein levels and attenuate electrophysiological abnormalities [47]。Parkin mutations are associated with most familial early-onset Parkinson's disease. parkin interacts with the n-terminus of ATXN2 to ubiquitinate normal and expanded ATXN2. parkin overexpression attenuates toxicity induced by ATXN2 expansion [47]. In some PD patients, the CAG repeat sequence of the ATXN2 gene is longer, which may be related to the development of PD. Treatments that target ATXN2 may help reduce the symptoms of PD or delay disease progression [47].ATXN2 plays multiple roles in neurological function and neurodegenerative diseases, including directly affecting neurological function through specific molecular and cellular pathways [52].Therefore, therapeutic strategies that target the complex functions of ATXN2 may be useful in the treatment of a wide range of neurodegenerative diseases.

ATXN2 is currently associated with a variety of diseases, but the specific mechanisms of its action in different diseases are not fully understood. For example, ATXN2 is associated with SCA2, but how it contributes to disease progression remains unknown [47].The binding properties and functions of ATXN2 as an RNA-binding protein to specific RNA sequences have not been fully elucidated, and the regulatory mechanisms by which ATXN2 is transcriptionally induced during starvation and involved in the cellular stress response [135],as well as how post-translational modifications [136],affect its function and its relationship to disease, still require in-depth understanding. The phase-separated properties of ATXN2 and its role in circadian rhythms have been studied to some extent in recent studies, but its role in other physiological or pathological processes remains unclear [44].

ATXN2 interacts with a wide range of proteins, but a comprehensive understanding of its interaction network and the role of these interactions in disease needs to be further investigated.ATXN2 was found to be immunologically relevant early on, but little research has been carried out in the area of immunity.ATXN2 is involved in tumour progression, but the specific mechanisms involved remain understudied in the field.ATXN2 has been found to be a major contributor to the development of tumours, but the specific mechanisms involved remain understudied.

This review has primarily focused on elucidating the exact roles of ATXN2 in cancer and its involvement in related cellular processes and signaling pathways. When combined with relevant research data, it leads to the hypothesis that different epigenetic modifications of ATXN2 may play distinct roles in various diseases, a hypothesis that warrants further investigation in clinical or experimental settings. Moreover, research into the role of ATXN2 in immunoregulation is limited, thus representing an area ripe for further exploration. From the intricate molecular interactions to the protein level, the multifaceted functions of ATXN2, along with its interactions with various molecules or proteins, offer promising directions for the development of disease treatments.

## Data Availability

Not applicable.
